# Food as Medicine: Effects of a Culinary Medicine Food-Demonstration on Medical Students’ Nutrition Knowledge, Confidence, and Satisfaction: An Exploratory Pilot Non-Randomized Pre-Post Intervention Study

**DOI:** 10.3390/nu18132203

**Published:** 2026-07-07

**Authors:** Niayesh Mardmomen, Cyrus Fatemi, Olivia K. Smith, Michael Roberts, Farzaneh Daghigh

**Affiliations:** The Department of Biomedical Sciences, Philadelphia College of Osteopathic Medicine, Philadelphia, PA 19131, USA; nm3696@pcom.edu (N.M.); cf3645@pcom.edu (C.F.); os3475@pcom.edu (O.K.S.); michaelrob@pcom.edu (M.R.)

**Keywords:** nutrition education, culinary medicine, medical school, curriculum

## Abstract

**Background**: Diet plays a central role in the development of chronic diseases, making nutrition-focused interventions a powerful tool for improving population health. However, substantial gaps in nutrition education persist in the U.S. medical education, and medical students frequently report receiving limited nutrition training despite its importance in patient care. **Methods**: A non-randomized pre-post intervention study with a one-month follow-up was conducted among osteopathic medical students. Participants attended a culinary medicine food-demonstration featuring five case-based topics and three chef-prepared recipes. Nutrition knowledge, satisfaction with nutrition education, confidence in nutrition counseling, and personal nutrition-related behaviors were assessed at baseline, immediately post-intervention, and one month later. Paired-samples t-tests with effect sizes (Cohen’s d) were used to compare scores over time. **Results**: Forty-six participants were assessed for nutrition knowledge and satisfaction, of whom forty-three completed a one-month follow-up in which confidence and behavior modification were evaluated. Nutrition knowledge significantly improved, with quiz scores increasing from 57.8 ± 13.8 to 75.2 ± 13.3 (*p* < 0.001, d = 1.04). Satisfaction with nutrition education and confidence in nutrition counseling also increased significantly (both *p* < 0.001), with a large effect size for confidence (d = 2.26). No significant change was observed in personal nutrition-related behaviors (*p* = 0.65, d = −0.06). Fifteen students who had previously completed a culinary medicine elective demonstrated higher baseline knowledge, satisfaction, and counseling confidence than students without prior training (all *p* ≤ 0.006), while nutrition-related behaviors remained largely unchanged in both groups. **Conclusions**: A brief culinary medicine food-demonstration session produced improvements in medical students’ nutrition knowledge, satisfaction with nutrition education, and confidence in nutrition counseling, and did not measurably change short-term personal nutrition behaviors. These findings support this model as an efficient approach to enhancing medical students’ nutrition-related competencies.

## 1. Introduction

Diet is a major driver in the development of many chronic diseases, making nutrition-focused interventions a powerful tool for improving population health and reducing disease burden, premature deaths, and healthcare costs [1]. There is evidence of large gaps in medical nutrition education and training in the United States, and medical students often report limited nutrition education, despite its central role in patient care [2,3]. Without practical, skills-based training, many future clinicians feel unprepared to translate nutrition guidelines into practical recommendations to address patients’ needs. Importantly, nutrition knowledge, counseling confidence, and personal dietary behaviors represent related but distinct domains: knowledge reflects understanding of evidence-based principles, confidence reflects perceived ability to apply this knowledge in patient care, and personal behavior reflects sustained lifestyle choices that are influenced by broader contextual and environmental factors.

Experiential interventions, including culinary medicine and food-demonstrations, have been proposed as effective strategies to improve knowledge, self-efficacy, and counseling confidence [4,5,6]. A large prospective cohort study of 4026 students at 45 U.S. medical schools found that those who received culinary medicine training, compared with matched peers in traditional curricula, performed better on 25 diet-related preventive cardiology competencies and also reported healthier personal eating habits [7]. Embedding structured culinary medicine experiences into undergraduate medical curricula could therefore bridge this gap by allowing students to practice applying evidence-based nutrition principles to real-world scenarios, ultimately strengthening their ability to deliver high-quality, diet-focused counseling in clinical practice.

Lifestyle habits established during training are also highly relevant, as medical students and other university populations often experience high levels of stress, suboptimal dietary patterns, and limited physical activity, all of which can negatively affect well-being and future professional practice. Guerriero et al. [8] highlight the interconnected nature of stress, lifestyle behaviors, and health outcomes in university students, emphasizing the need for integrated educational approaches that simultaneously target knowledge, behaviors, and well-being. Such findings underscore the importance of addressing not only students’ competencies in counseling patients, but also their own health behaviors as emerging healthcare professionals.

There are limited data on the impact of brief, single-session culinary medicine interventions within required medical curricula, particularly in osteopathic training programs. Given how dense medical school curricula already are, evaluating the effectiveness of short, focused sessions represents an important and practical next step.

The objective of this exploratory pilot study was to evaluate the impact of a one-hour culinary medicine food-demonstration on medical students’ nutrition knowledge, satisfaction with nutrition education, confidence in counseling patients, and personal nutrition behaviors. We hypothesized that the intervention would improve nutrition knowledge, students’ satisfaction and confidence, while short-term behavior change might be limited.

## 2. Materials and Methods

We conducted a non-randomized pre-post intervention study with a one-month follow-up to assess the effects of a culinary medicine food-demonstration session on medical students’ nutrition-related outcomes. Outcomes were measured at baseline (pre-intervention), immediately after the demonstration (post-intervention), and one month later. No changes were made to the study protocol, outcomes, or procedures after data collection began. Due to the nature of the educational intervention, the blinding of participants, instructors, or outcome assessors was not feasible.

**Figure 1:**
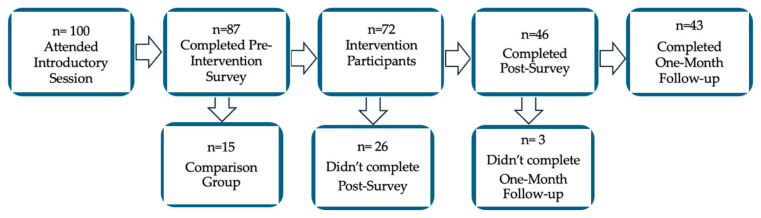
Participant flow diagram. Participant attrition was observed at each stage of the study. The comparison group (*n* = 15) consisted of students who had previously completed the Culinary Medicine elective and completed the pre-survey only.

Participants were second-year medical students at Philadelphia College of Osteopathic Medicine, Philadelphia, PA, USA (PCOM). Students were informed of the voluntary introductory session via announcements and attended on a self-selected basis outside of required coursework. All students had equal access to the introductory session. A total of 100 students attended the introductory session; 87 completed the pre-intervention survey. Of which fifteen students had previously completed the Culinary Medicine elective. This group was included as a comparison group but were excluded from participating in the intervention. 72 students were invited to attend the culinary medicine food-demonstration session (Intervention). Among them, 46 (31 women and 15 men) completed the post-intervention survey and 43 (31 women and 12 men) completed the one-month follow-up (Figure 1). Participants were aged 22–36 years. Thirty-five held a bachelor’s degree and 11 held a master’s degree (33 bachelor’s degrees and 10 master’s degrees who completed a one-month survey) (Table 1). Data collection occurred across two cohorts. For Cohort 1, the introductory session and pre-survey were administered on 28 July 2025, with food-demonstration sessions and post-intervention surveys completed on 1–2 October 2025. The one-month follow-up survey was distributed on 7 November 2025, with a one-week completion window (closed 14 November 2025). For Cohort 2, the introductory session and pre-survey were administered on 7 October 2025, with the food-demonstration and post-intervention survey completed on 29 October 2025. The one-month follow-up survey was distributed on 3 December 2025, with a one-week completion window (closed 10 December 2025). Nutrition knowledge and satisfaction were assessed immediately after the intervention, with statistical analyses conducted on 46 participants who attended the culinary demonstration. Subsequently, a one-month follow-up phone call assessed participants’ confidence in nutrition counseling and behavior modification. Of the original participants, forty-three completed this follow-up; therefore, analyses for confidence and behavior changes are based on *n* = 43 (Figure 1). A gift card was offered as an incentive to encourage attendance and survey completion. Participation was entirely voluntary. 

The culinary medicine food-demonstration sessions were conducted in a structured culinary medicine case-based format, designed to provide practical nutrition education and model healthy cooking techniques. The sessions featured five patient cases, each focused on a different topic: the Mediterranean Diet, the Power of Legumes, Anti-inflammatory Foods for Disease Prevention, Soluble and Insoluble Fibers and Helping Patients Make Informed Food Choices. The educational objectives for these cases were to identify key nutrition concepts, recognize relevant food sources, and apply evidence-based dietary guidance in patient counseling. The educational objectives for the Mediterranean Diet case were to identify the main components of the Mediterranean diet and its relevance to health promotion. For the Power of Legumes case, the objectives were to recognize legumes as a source of plant-based protein and dietary fiber. For the Anti-inflammatory Foods for Disease Prevention case, the objectives were to identify foods commonly associated with anti-inflammatory dietary patterns and disease prevention. For the Soluble and Insoluble Fibers case, the objectives were to differentiate between soluble and insoluble fiber and their dietary sources. For the Helping Patients Make Informed Food Choices case, the objective was to apply basic strategies to help patients with Type-2 diabetes make informed food choices tailored to their disease. These interactive sessions effectively engaged students in understanding core nutrition concepts, applying nutrition knowledge to patient counseling, and opening discussion revolving around healthy cooking practices.

During the culinary medicine food-demonstration sessions, the assigned chef developed and demonstrated three recipes, which the students were able to taste during the session: a white bean dip with veggies, olive oil lemon quinoa salad, and sunflower butter granola protein balls. The chef arrived at the beginning of the session, completed simple preparation steps with ingredients partially prepped in advance, and the demonstrations took approximately 10–15 min. These nutritious recipes were intentionally aligned with the patient cases in order to show the study participants their nutritional value, how to prepare them, and offer practical methods to recommend these recipes to their future patients [1,9]. The educational case presentations were delivered while students consumed the food, allowing the culinary demonstrations, educational and counseling messages to be integrated into a single interactive experience. Students completed the post-quiz assessment during the final 10 min of the session. Nutrition knowledge was assessed using a Nutrition Education Quiz administered at the introductory session and again after the culinary medicine food-demonstration; that is, the pre- and post-intervention surveys (Table 2). The Nutrition Education Quiz with answer options may be accessed (Table S1). Satisfaction with current nutrition education (Table 3), confidence in nutrition counseling (Table 4), and personal nutrition-related behaviors (Table 5) were measured using Likert-type survey scales at baseline and follow-up time points. All three surveys were developed by the study investigators.

Nutrition knowledge was assessed using a 10-item Nutrition Education Quiz administered at the introductory session and again immediately after the culinary medicine food-demonstration. Each item was multiple choice with one correct answer, and the total score was calculated as the percentage of correct responses (range 0–100%), with higher scores indicating greater nutrition knowledge. Satisfaction with nutrition education was measured using a 6-item Likert-type scale (Table 3). Items were rated from 1 (“not at all satisfied”) to 5 (“very satisfied”), and responses were summed to yield a total satisfaction score ranging from 6 to 30, with higher scores reflecting greater satisfaction with nutrition education. Confidence in nutrition counseling was measured using a 5-item Likert-type scale (Table 4). Items were rated from 1 (“not at all confident”) to 5 (“strongly confident”), and summed to create a total confidence score ranging from 5 to 25, with higher scores indicating greater confidence in providing nutrition counseling. Personal nutrition-related behaviors were measured using a 5-item frequency scale (Table 5). Items were rated from 1 (“never”) to 5 (“daily”), and responses were summed to generate a total behavior score ranging from 5 to 25, with higher scores representing more frequent engagement in nutrition promoting behaviors (e.g., intake of fruits, vegetables, whole grains, and legumes, and more frequent home cooked meals). No formal assessment of internal consistency or other properties was performed for these investigator-developed scales.

Descriptive statistics (means and standard deviations) were calculated for all continuous outcome measures at each time point. Normality of pre–post and baseline–follow-up difference scores for each outcome was evaluated using the Shapiro–Wilk test. Satisfaction difference scores approximated normality, whereas difference scores for the Nutrition Education Quiz, confidence in nutrition counseling, and personal nutrition-related behaviors deviated from normality. Primary analyses used paired-samples t-tests to compare pre- and post-intervention scores, as well as baseline and one-month follow-up scores. For outcomes with non-normal difference scores (quiz, confidence, and behavior), sensitivity analyses using Wilcoxon signed-rank tests were conducted; these yielded conclusions consistent with the parametric tests. Independent-samples t-tests (or Welch’s t-test when variances were unequal) were used to compare baseline outcomes between students who had previously completed the culinary medicine elective and those who had not. Effect sizes were calculated using Cohen’s d, and 95% confidence intervals for the mean differences were computed. Analyses were conducted on a per-protocol basis, including only participants who completed assessments at each respective time point; no intention-to-treat analysis was performed, and no imputation was performed for missing data. An a priori power analysis was conducted to estimate the required sample size. Given the planned paired-samples *t*-tests and independent-samples *t*-tests (four comparisons each), a Bonferroni correction was applied, yielding an adjusted alpha level of 0.0125 (0.05 ÷ 4). Assuming a moderate effect size, α = 0.0125, and minimum acceptable power of 0.80, the paired-samples analyses required a minimum of 41 participants, while the independent-samples analyses required a minimum of 156 participants. The achieved sample sizes of *n* = 43–46 for paired analyses met this threshold; however, the independent-samples comparisons were underpowered given the available comparison group sample of *n* = 15. All statistical analyses were performed using IBM SPSS version 28.

## 3. Results

Participants reported varying levels of prior nutrition education, including none (*n* = 1), High School-level nutrition (*n* = 17), undergraduate nutrition courses (*n* = 13), graduate-level nutrition coursework (*n* = 2), and nutrition education during medical school (*n* = 13). Assessments of nutrition knowledge, satisfaction with the intervention, confidence in nutrition counseling of patients, and personal nutrition-related behavioral changes were conducted and outcomes are summarized in Table 6.

**Figure 2:**
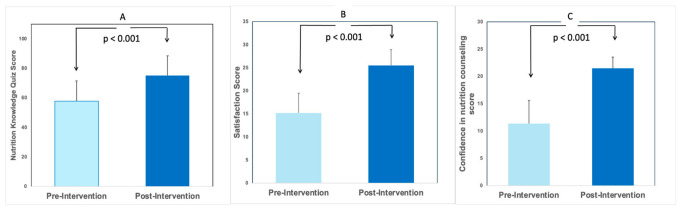
Assessments conducted before and after the culinary medicine food-demonstration (pre- and post-intervention, respectively) included (**A**) mean nutrition knowledge quiz scores (*n* = 46), (**B**) mean satisfaction with the intervention (*n* = 46), and (**C**) mean confidence in counseling patients about nutrition (*n* = 43). Error bars represent standard errors in (**A**–**C**). Scores increased significantly following all three measures (*p* < 0.001).

**Figure 3:**
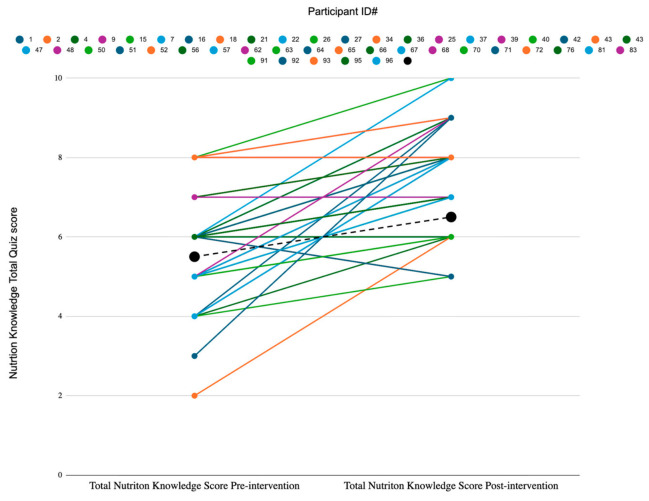
Individual changes in nutrition knowledge quiz scores from baseline (pre-intervention) to post-intervention, *n* = 46. Participants with identical scores are represented by a single line. The quiz was scored from 0 to 10, with one point awarded for each correct response. The pre-and post-intervention averages are connected with a black dashed line.

Nutrition Knowledge assessment (*n* = 46): The mean Nutrition Education Quiz score prior to the culinary food-demonstration was 57.8 (SD = 13.8) and increased to 75.2 (SD = 13.3) after the session, Figure 2A. A paired-samples *t*-test revealed a statistically significant increase in Nutrition Education Quiz scores from pre- to post-intervention, *t* (45) = 7.08, *p* < 0.001 with a large effect size (Cohen’s d = 1.04). The 95% confidence interval for the mean difference ranged from 12.44 to 22.34. A paired individual-level plot to show within-participant changes and variability is displayed for nutrition knowledge for pre- and post-intervention in Figure 3.

Assessment of satisfaction with the intervention (*n* = 46): The mean satisfaction score increased from 15.2 (SD = 4.3) at baseline to 25.5 (SD = 3.5) post-intervention, Figure 2B. A paired-samples *t*-test indicated a statistically significant increase in satisfaction scores before the food-demo session and after the food-demo session, *t* (45) = 14.38, *p* < 0.001, with a very large effect size (Cohen’s d = 2.12).

Confidence in nutrition counseling of patients (*n* = 43): The mean student’s confidence in nutrition counseling score increased from 11.4 (SD = 4.2) at baseline to 21.5 (SD = 2.1) at the one-month follow-up, Figure 2C. A paired-samples *t*-test indicated a statistically significant increase in students’ confidence in nutrition counseling scores between the two time points, *t* (42) = 14.83, *p* < 0.001, with a very large effect size (Cohen’s d = 2.26).

Personal nutrition-related behavioral changes (*n* = 43): The mean behavioral change score before the culinary medicine food-demonstration intervention was 16.42 (SD = 2.76), and the mean score after the food-demonstration was 16.28 (SD = 2.14). A paired-samples *t*-test indicated no statistically significant difference in personal behavioral change scores between the two time points, *t* (42) = −0.41, *p* = 0.684, and the effect size (Cohen’s d = −0.06) indicated a negligible effect.

Comparison between the intervention group and comparison group: The comparison group of fifteen students who had previously taken the culinary medicine elective at PCOM completed the pre-intervention survey; their data were compared with that of 72 students who had not taken the elective. The mean nutrition quiz score of students who had never taken the culinary medicine elective was 58.19 (SD = 12.71), whereas the mean score for students who had taken the culinary medicine elective was 70.00 (SD = 13.09). An independent-samples *t*-test indicated a statistically significant difference in nutrition quiz scores between the two groups, *t* (85) = 3.26, *p* = 0.002, with a large effect size (Cohen’s d = 0.92). These results indicate that students who had taken the culinary medicine elective demonstrated greater nutrition knowledge than those who had not. The mean satisfaction score for the intervention group was 15.17 (SD = 3.98), compared with 19.60 (SD = 5.580 for the comparison group. An independent-samples *t*-test showed a statistically significant difference between the two groups *t* (85) = 3.64, *p* < 0.001, with a large effect size (Cohen’s d = 1.03). These results indicate that the comparison group reported substantially higher satisfaction with the nutrition education than the intervention group. The mean confidence score for nutrition counseling was lower in the intervention groups 11.01 (SD = 3.69) compared to the comparison group 15.80 (SD = 5.67). Because the assumption of equal variances was not met, an independent-samples *t*-test with unequal variances (Welch’s test) was used. The results indicated a statistically significant difference in confidence scores between the two groups, *t* (16.56) = 3.13 and *p* = 0.006, with a large effect size (Cohen’s d = 1.17). These findings suggest that students in the comparison group were markedly more confident in nutrition counseling than those in the intervention group.

Correlation between the comparison group and intervention group in personal nutrition-related behavioral changes: The mean oftenness score of students who had never taken the culinary medicine elective was 16.21 (SD = 2.61), and for those who had taken the culinary medicine elective it was 16.20 (SD = 3.57). An independent-samples *t*-test indicated no statistically significant difference between the two groups, *t* (85) = −0.01 and *p* = 0.992, and the effect size (Cohen’s d ≈ 0) indicated a negligible difference in behavior. Therefore, participation in the culinary medicine elective was not associated with any meaningful change in self-reported nutrition-related behaviors. No adverse events or unintended effects were observed.

## 4. Discussion

The findings of this study indicate that a brief, food-demonstration-based nutrition education session was associated with meaningful improvements in medical students’ nutrition knowledge, satisfaction with nutrition education, and confidence in nutrition-related decision-making. Knowledge scores increased substantially following the intervention, with a large effect size, suggesting that even a single structured culinary medicine food-demonstration may be associated with measurable educational gains [2,3,6]. Participants also reported markedly higher satisfaction with nutrition education after the session, reflecting strong engagement and perceived value of the food-demonstration format [6]. These results are consistent with prior studies demonstrating that experiential nutrition education, including culinary medicine food-demonstration models, enhances learning outcomes in medical and health professional training [2,6]. Culinary medicine programs integrate didactic nutrition science with hands-on skill development, allowing learners to practice counseling strategies in realistic, applied contexts [5,6]. Such models have been proposed as vital for equipping future clinicians with practical knowledge and skills to support patients’ dietary behavior changes, which is critical for chronic disease prevention and management [2,3]. By explicitly modeling evidence-based nutrition counseling and simple, replicable recipes, the session in this study may have served as a behavior-modeling experience that linked didactic content to practical clinical conversations and everyday food choices, and reinforced learners’ perceptions of the importance of nutrition in clinical care.

These findings align with prior work suggesting that while brief educational interventions can produce immediate gains in nutrition knowledge and counseling confidence, sustained improvements in personal dietary behaviors likely require longitudinal, integrated approaches that address the broader lifestyle context of students, including stress and well-being [8]. A one-hour session may be sufficient to improve awareness, engagement, and confidence, but it is unlikely to produce durable habit change without repeated exposure, goal-setting, environmental reinforcement, and longer follow-up.

The increase in satisfaction scores aligns with previous observations that interactive, applied nutrition education tends to be more engaging than traditional lecture-based formats [9,10]. Self-efficacy gains maintained at the one-month follow-up suggest that the curriculum may have helped strengthen participants’ confidence in making nutrition-related decisions and discussing nutrition with patients [9,11]. Similar sustained confidence increases have been reported in prior studies of medical students and other health professionals following experiential nutrition education interventions [9,11]. The effectiveness of the curriculum was reflected not only in knowledge acquisition but also in learners’ increased confidence in discussing nutrition with patients.

The comparison group demonstrated significantly higher nutrition knowledge, satisfaction with nutrition education, as well as greater confidence in nutrition counseling than those who had not. These patterns are consistent with evidence that longer exposure to culinary medicine can substantially enhance learners’ competence and attitudes toward nutrition education. Moreover, the large within-group increase in confidence from baseline to one-month follow-up in the intervention cohort suggests that even a single focused culinary medicine food-demonstration may still provide meaningful benefit for students without prior elective experience.

Despite improvements in knowledge and self-efficacy, reported personal health behaviors did not change significantly between baseline and one-month follow-up. This finding is consistent with evidence that cognitive and psychosocial outcomes, such as knowledge, attitudes, and self-efficacy, often improve in the short term, whereas observable changes in dietary behavior typically require sustained engagement and reinforcement beyond initial training [5,9,11]. Behavior changes frameworks, such as PRECEDE-PROCEED, highlight that knowledge and confidence are necessary but not sufficient for lasting behavioral change. Enabling and reinforcing factors including social support, access to healthy foods, and ongoing practice, are essential for translating educational gains into habitual behaviors [11].

Teaching kitchens and Food as Medicine programs frequently integrate motivational strategies, interactive goal-setting, and personalized support to address these barriers, emphasizing the importance of multi-component intervention designs for promoting behavior change [5,6].

Several limitations should be considered. This study was conducted at a single institution, which may limit generalizability to other settings and populations. The study also had a small sample size, which may limit statistical power and increase the risk of Type II error. Additionally, the survey instruments were investigator-developed and were not previously validated for this population or context, and no formal psychometric evaluation (e.g., internal consistency or construct validity) was performed. This limits the precision of the measures and may affect the generalizability of the findings. The absence of randomization and the lack of a true control group also limit the ability to draw causal inferences about the intervention’s effects. The comparison group’s analyses were underpowered due to the limited size of the prior elective completion cohort (*n* = 15), and the between-group findings should be interpreted as exploratory. The one-month follow-up may be insufficient to measure meaningful changes in dietary behavior, which often emerge over longer periods [9]. Self-reported measures of behavior and confidence are susceptible to social desirability and recall bias [4]. The self-selected nature of participation, in which students voluntarily attended a session outside of required coursework, may limit generalizability, as attendees likely had pre-existing interest in nutrition, potentially inflating observed outcomes [2,5]. While the study focused on educational outcomes, it did not include objective measures such as dietary intake tracking or biomarkers, which could have offered additional insights into behavior change [6]. There was also no assessment of actual counseling performance, which would provide a more direct measure of clinical skill development. The study did not explicitly address potential attrition between baseline and follow-up, which could influence the observed outcomes. Despite these limitations, this exploratory pilot study provides valuable insights into the potential of brief, culinary medicine food-demonstration-based nutrition education, and the findings should not be overgeneralized beyond the studied population.

## 5. Conclusions

This study suggests that a single, one-hour, case-based culinary medicine food-demonstration delivered in a non-specialized space was associated with meaningful improvements in medical students’ nutrition knowledge, satisfaction with nutrition education, and confidence in nutrition counseling, with no significant short-term change in personal nutrition-related behaviors. The present support the feasibility and potential educational value of culinary medicine food-demonstration-based education as a low-burden, low-cost strategy that could be adapted across medical schools, including those without full teaching kitchens. While short-term behavioral change was not observed, the intervention may have contributed to greater readiness for future engagement in healthier behaviors [2,6]. Future research should evaluate multi-session and longitudinal implementations, randomization, incorporate objective measures of dietary behavior, examine downstream clinical impact on patient counseling and health indicators, and explore strategies for integrating culinary medicine vertically across osteopathic curricula to maximize their impact at both learner and patient levels [5,9,12].

## Figures and Tables

**Table 1:** Baseline demographic characteristics of study participants by group.

Variable	Response Option	Completed Nutrition Knowledge & Satisfaction *n* = 46	Completed All Assessments *n* = 43	Comparison Group *n* = 15
Gender	Women	31	31	9
	Men	15	12	6
Education Degree	Bachelor	35	33	9
	Master’s	11	10	6
Age Range	22–29	42	40	14
	30–36	4	3	1

**Table 2:** Nutrition Education Quiz questions. The response options for these questions were multiple choice options.

Item	Nutrition Education Quiz Questions
1	Which of the following lists the hallmarks of the Mediterranean diet?
2	Which of the following is a source of dietary fat?
3	What are the health benefits of legumes?
4	What are the health benefits of quinoa?
5	Which dietary pattern is most strongly associated with a higher risk of colorectal cancer?
6	Which of the following is least associated with anti-inflammatory effects in the human body?
7	In managing hypertension, the DASH diet is thought to lower blood pressure primarily through which strategy?
8	Which of the following clinical benefits is most consistently associated with long-term adherence to the Mediterranean diet in adults?
9	A 75-year-old woman with a history of breast cancer and chronic kidney disease presents with fatigue and muscle weakness. Laboratory tests reveal low serum 25-hydroxy vitamin D levels. Considering her medical history, what is the primary reason to recommend vitamin D supplementation?
10	A 32-year-old woman with cystic fibrosis and a history of pancreatic insufficiency presents with worsening fatigue, muscle weakness, and occasional difficulty with balance. She admits to poor adherence to her vitamin supplements. On exam, she has mild hyporeflexia in the lower limbs. Which fat-soluble vitamin deficiency is most likely contributing to her symptoms?

**Table 3:** Satisfaction with current nutrition education questions. The response options were: Very satisfied, satisfied, moderately satisfied, somewhat satisfied, not at all satisfied.

Item	Satisfaction in Nutrition Education Questions
1	How satisfied are you with the nutrition education you have received in medical school so far?
2	How satisfied do you feel in your nutrition knowledge currently when it comes to discussing nutritious foods?
3	How satisfied are you in your nutrition knowledge, based on your current medical training, in providing nutrition guidance to patients?
4	How satisfied do you feel with your preparedness to give patients basic advice about healthy eating?
5	How familiar are you with the Mediterranean diet and its clinical benefits?
6	Do you agree that nutrition education is currently meaningfully integrated into your medical training?

**Table 4:** Confidence in nutrition counseling questions. The response options were: Strongly confident, confident, moderately confident, slightly confident, not at all confident.

Item	Confidence in Nutrition Counseling Statement
1	How confident do you feel providing patients with evidence-based nutrition advice?
2	How confident do you feel discussing dietary changes with patients to support their health goals?
3	How confident do you feel advising patients on incorporating plant-based proteins into their diets?
4	How confident do you feel explaining the benefits of the Mediterranean diet to patients?
5	How confident do you feel about your current knowledge of nutritional guidelines and dietary recommendations relevant to patient care?

**Table 5:** Personal nutrition-related behaviors questions. The response options were: (Never, 1–3 times/month, 1–3 times/week, 4–6 times/week, Daily, I do not like whole grains, I have an allergy to whole grain).

Item	Nutrition-Related Behavior Questions
1	How often do you eat fruits and vegetables?
2	How often do you eat whole grains (such as whole wheat cereals, quinoa, brown rice, oats, Farro, or barley)?
3	How often do you eat legumes (nuts, beans or peas)?
4	How often do you eat fast food or ultra-processed food weekly?
5	How often do you eat home cooked meals weekly?

**Table 6:** Outcome assessments. Assessments were administered at baseline (pre-intervention), immediately after the intervention (post-intervention), and at one-month follow-up.

Outcome Assessment	Pre-Intervention	Post-Intervention	One-Month Follow-Up	*n*	df	Test Statistic	*p*-Value
Knowledge	X	X		46	45	7.08	0.001
Satisfaction	X	X		46	45	14.38	0.001
Confidence	X		X	43	42	14.83	0.001
Behavior	X		X	43	42	−0.41	0.684

## Data Availability

Data described in the manuscript, the codebook, and analytic code will be made available upon reasonable request and approval due to privacy and ethical reasons.

## Supplementary Materials

The following supporting information can be downloaded at: https://www.mdpi.com/article/10.3390/nu18132203/s1, Table S1: Nutrition Education Questions and Answer Options.

## References

[1] Gropper S.S. (2023). The Role of Nutrition in Chronic Disease. Nutrients.

[2] Polak R., Phillips E.M., Nordgren J., La Puma J., La Barba J., Cucuzzella M., Graham R., Harlan T., Burg T., Eisenberg D. (2016). Health-related Culinary Education: A Summary of Representative Emerging Programs for Health Professionals and Patients. Glob. Adv. Health Med..

[3] Adams K.M., Kohlmeier M., Zeisel S.H. (2010). Nutrition education in U.S. medical schools: Latest update of a national survey. Acad. Med..

[4] Althubaiti A. (2016). Information bias in health research: Definition, pitfalls, and adjustment methods. J. Multidiscip. Healthc..

[5] Monlezun D.J., Dart L., Vanbeber A., Smith-Barbaro P., Costilla V., Samuel C., Terregino C.A., Abali E.E., Dollinger B., Baumgartner N. (2018). Machine Learning-Augmented Propensity Score-Adjusted Multilevel Mixed Effects Panel Analysis of Hands-On Cooking and Nutrition Education versus Traditional Curriculum for Medical Students as Preventive Cardiology: Multisite Cohort Study of 3,248 Trainees over 5 Years. BioMed Res. Int..

[6] Eisenberg D.M., Pacheco L.S., McClure A.C., McWhorter J.W., Janisch K., Massa J. (2023). Perspective: Teaching Kitchens: Conceptual Origins, Applications and Potential for Impact within Food Is Medicine Research. Nutrients.

[7] Sacks F.M., Lichtenstein A.H., Wu J.H.Y., Appel L.J., Creager M.A., Kris-Etherton P.M., Miller M., Rimm E.B., Rudel L.L., Robinson J.G. (2017). Dietary Fats and Cardiovascular Disease: A Presidential Advisory from the American Heart Association. Circulation.

[8] Guerriero M.A., Dipace A., Monda A., De Maria A., Polito R., Messina G., Monda M., di Padova M., Basta A., Ruberto M. (2025). Relationship Between Sedentary Lifestyle, Physical Activity and Stress in University Students and Their Life Habits: A Scoping Review with PRISMA Checklist (PRISMA-ScR). Brain Sci..

[9] Crowley J., Ball L., Laur C., Wall C., Arroll B., Poole P., Ray S. (2015). Nutrition guidelines for undergraduate medical curricula: A six-country comparison. Adv. Med. Educ. Pract..

[10] Harkin N., Johnston E., Mathews T., Guo Y., Schwartzbard A., Berger J., Gianos E. (2018). Physicians’ Dietary Knowledge, Attitudes, and Counseling Practices: The Experience of a Single Health Care Center at Changing the Landscape for Dietary Education. Am. J. Lifestyle Med..

[11] Scott C.M. (2001). Health Promotion Planning: An Educational and Ecological Approach (3rd ed). Can. J. Public Health.

[12] Mozaffarian D., Aspry K.E., Garfield K., Kris-Etherton P., Seligman H., Velarde G.P., Williams K., Yang E. (2024). “Food Is Medicine” Strategies for Nutrition Security and Cardiometabolic Health Equity: JACC State-of-the-Art Review. J. Am. Coll. Cardiol..

