# Supplementary material for: Food as Medicine: Effects of a Culinary Medicine Food-Demonstration on Medical Students’ Nutrition Knowledge, Confidence, and Satisfaction: An Exploratory Pilot Non-Randomized Pre-Post Intervention Study
Source: Nutrients. 2026 Jul 7;18(13):2203. doi: 10.3390/nu18132203 (PMC13364193; doi:10.3390/nu18132203)
Supplement: Supplementary file 1 [file nutrients-18-02203-s001.zip › nutrients-4360540-supplementary.pdf]

Nutrition Education Questions and Answer Options

1. Which of the following lists the hallmarks of the Mediterranean diet?
    a) High intake of fruits and vegetables/ moderate intake of fish, seafood and dairy, low intake of red and processed meats
    b) Moderate intake of fruits and vegetables/ low intake of fish, seafood and dairy/ high intake of red and processed meats
    c) Low intake of fruits and vegetables/ high intake of fish, seafood and dairy/ moderate intake of red and processed meats

    Correct answer: a is correct. Rational: The Mediterranean diet emphasizes high intake of fruits, vegetables, legumes, whole grains, olive oil, nuts, moderate fish and dairy (especially yogurt and cheese), and low red/processed meat.

2. Which of the following is a source of dietary fat?
    a) Banana
    b) Avocado
    c) Brown Rice
    d) Spinach
    e) Chickpeas

    Correct answer: b is correct. Rational: Avocado is naturally rich in monounsaturated fats. Banana, brown rice, and spinach contain negligible fat. Chickpeas contain only a small amount (~2–3% by weight) and are not considered a dietary fat source.

3. What are the health benefits of legumes?
    a) They directly modulate cholesterol absorption.
    b) They provide a complete source of all essential amino acids.
    c) They increase LDL cholesterol levels due to their high phytosterol concentration.
    d) They improve gut microbiota diversity through fermentable fibers and oligosaccharides.
    e) They enhance Vitamin B_{12} status due to their high cobalamin content.

    Correct answer: d is correct. Rational: Legumes contain fermentable fibers and oligosaccharides that nourish beneficial gut bacteria, improving microbiota diversity and production of short-chain fatty acids.

4. What are the health benefits of quinoa?
    a) It provides amino acid profile, excluding lysine and methionine.
    b) It significantly reduces heme iron absorption due to its high phytic acid content.
    c) It contributes to cardiovascular health through its high omega-3 fatty acid content.
    d) It supports antioxidant defense mechanisms via polyphenols and flavonoids.
    e) It promotes postprandial glucose spikes due to its high glycemic index.

Correct answer: d is correct. Rational: Quinoa contains polyphenols and flavonoids with antioxidant activity. It is also a complete protein. Option (a) is clearly false because quinoa contains both lysine and methionine. (b), (c), and (e) are false.

5. Which dietary pattern is most strongly associated with a higher risk of colorectal cancer?
   a) High intake of fiber-rich whole grains and vegetables
   b) Frequent consumption of sandwiches with deli meats
   c) Predominantly plant-based diet with limited animal products
   d) Regular consumption of fish and poultry as main protein sources
   e) Increased intake of fermented dairy products such as yogurt.

   Correct answer: b is correct. Rational: Frequent intake of processed meats (such as deli meats) is consistently associated with increased colorectal cancer risk and is classified as carcinogenic to humans.

6. Which of the following is least associated with anti-inflammatory effects in the human body?
   a) Curcumin from turmeric, due to its inhibition of NF-κB signaling
   b) Omega-3 fatty acids, through modulation of eicosanoid pathways
   c) Anthocyanins in berries, which suppress pro-inflammatory cytokines
   d) Trans fats, through enhancement of systemic oxidative stress
   e) Epigallocatechin gallate (EGCG) in green tea, via COX-2 inhibition

   Correct answer: d is correct. Rational: Trans fats are pro-inflammatory, increasing oxidative stress and inflammatory signaling rather than reducing inflammation.

7. In managing hypertension, the DASH diet is thought to lower blood pressure primarily through which strategy? By:
   a) Limiting carbohydrates to reduce insulin resistance and vascular stiffness
   b) Increasing saturated fat to improve HDL levels and endothelial function
   c) Promoting protein from animal sources to suppress the renin-angiotensin system
   d) Emphasizing monounsaturated fats to decrease arterial wall tension
   e) Increasing consumption of potassium, magnesium, calcium, and reducing sodium intake.

   Correct answer: e is correct. Rational: The DASH diet lowers blood pressure primarily because it increases intake of potassium, magnesium, calcium, fiber, and lowers sodium intake.

8. Which of the following clinical benefits is most consistently associated with long-term adherence to the Mediterranean diet in adults?
   a) Improving glycemic control primarily through high protein intake
   b) Reducing risk of cardiovascular disease due to its overall dietary high intake of monounsaturated fats
   c) Prevention of iron-deficiency anemia through frequent consumption of red meat
   d) Lowering risk of chronic kidney disease via sodium restriction
   e) Decreasing bone fracture risk due to high dairy consumption

Correct answer: b is correct. Rational: Long-term Mediterranean diet adherence consistently reduces cardiovascular disease risk through healthy fats (especially monounsaturated fats from olive oil), plant foods, and anti-inflammatory effects.

9. A 75-year-old woman with a history of breast cancer and chronic kidney disease presents with fatigue and muscle weakness. Laboratory tests reveal low serum 25-hydroxy Vitamin D levels. Considering her medical history, what is the primary reason to recommend Vitamin D supplementation? To:
   a) enhance immune function and reduce cancer recurrence
   b) manage mineral and bone disorder from kidney disease
   c) improve muscle strength unrelated to kidney function
   d) prevent chemotherapy-induced neuropathy
   e) lower blood pressure in elderly patients

   Correct answer: b is correct. Rational: In chronic kidney disease, vitamin D supplementation is recommended to help prevent and manage chronic kidney disease–mineral and bone disorder (CKD-MBD), reducing secondary hyperparathyroidism and bone complications.

10. A 32-year-old woman with cystic fibrosis and a history of pancreatic insufficiency presents with worsening fatigue, muscle weakness, and occasional difficulty with balance. She admits to poor adherence to her vitamin supplements. Physical exam reveals that she has mild hyporeflexia in the lower limbs. Which fat-soluble vitamin deficiency is most likely contributing to her symptoms?
    a) Vitamin A
    b) Vitamin C
    c) Vitamin D
    d) Vitamin E
    e) Vitamin K

    Correct answer: d is correct. Rational: Cystic fibrosis with pancreatic insufficiency causes fat malabsorption. Vitamin E deficiency produces peripheral neuropathy, muscle weakness, ataxia, and hyporeflexia.

Answer Key

1. **a**
2. **b**
3. **d**
4. **d**
5. **b**
6. **d**
7. **e**
8. **b**
9. **b**
10. **d**
